# Presentation of antigen on extracellular vesicles using transmembrane domains from viral glycoproteins for enhanced immunogenicity

**DOI:** 10.1002/jev2.12199

**Published:** 2022-03-01

**Authors:** Kai Hu, Paul F. McKay, Karnyart Samnuan, Adrian Najer, Anna K. Blakney, Junyi Che, Gwen O'Driscoll, Martina Cihova, Molly M. Stevens, Robin J. Shattock

**Affiliations:** ^1^ Department of Infectious Diseases Imperial College London London UK; ^2^ Department of Materials Department of Bioengineering and Institute of Biomedical Engineering Imperial College London London UK; ^3^ Division of Radiotherapy and Imaging The Institute of Cancer Research London UK

**Keywords:** extracellular vesicle, immunogenicity, transmembrane domain, viral glycoprotein

## Abstract

A vaccine antigen, when launched as DNA or RNA, can be presented in various forms, including intracellular, secreted, membrane‐bound, or on extracellular vesicles (EVs). Whether an antigen in one or more of these forms is superior in immune induction remains unclear. In this study, we used GFP as a model antigen and first compared the EV‐loading efficiency of transmembrane domains (TMs) from various viral glycoproteins, and then investigated whether EV‐bound GFP (EV‐GFP) would enhance immune induction. Our data showed that GFP fused to viral TMs was successfully loaded onto the surface of EVs. In addition, GFP‐bound EVs were predominantly associated with the exosome marker CD81. Immunogenicity study with EV‐GFP‐producing plasmids in mice demonstrated that antigen‐specific IgG and IgA were significantly increased in EV‐GFP groups, compared to soluble and intracellular GFP groups. Similarly, GFP‐specific T cell response‐related cytokines produced by antigen‐stimulated splenocytes were also enhanced in mice immunized with EV‐GFP constructs. Immunogenicity study with purified soluble GFP and GFP EVs further confirmed the immune enhancement property of EV‐GFP in mice. In vitro uptake assays indicated that EV‐GFP was more efficiently taken up than soluble GFP by mouse splenocytes and such uptake was B cell preferential. Taken together, our data indicate that viral TMs can efficiently load antigens onto the EV surface, and that EV‐bound antigen enhances both humoral and cell‐mediated antigen‐specific responses.

## 1 INTRODUCTION

Vaccination represents one of the most effective approaches in the fight against infectious diseases. Traditionally, early viral vaccines were based on live‐attenuated or inactivated viruses, and while effective, often caused adverse effects (Amanna et al., 2012; Rappuoli, 2004). In recent years, the focus has shifted to the development of alternative safe and effective vaccine platforms, including those based on subunit proteins, nanoparticles and nucleic acids (Gerritzen et al., 2017; Kim et al., 2020; Porter & Raviprakash, 2016). While the newly developed platforms in general offer significantly improved safety, they suffer from other limitations like low immunogenicity (Guo et al., 2017; Suschak et al., 2017). The antigen(s) is the key component in a vaccine, which is responsible for the induction of antigen‐specific immune responses. Design and/or optimization of an antigen to improve immunogenicity is one of the most promising strategies to enhance the efficacy of a vaccine.

Nucleic acid vaccine platforms (e.g., DNA and RNA platforms) are easy to design and manufacture, leading to shortened development time and low‐cost vaccines (Kim et al., 2020). When a vaccine is launched via nucleic acid platforms, the antigen, depending on the design of the vaccine, could potentially be expressed intracellularly, secreted, or expressed on the cell surface. Alternatively, it can be designed for expression inside or on the surface of extracellular vesicles (EVs). The antigen in these different presentation forms has different characteristics and is likely to be directed to and exposed to different microenvironments. For instance, secreted and EV‐associated forms can travel remotely from the site of production to immune inductive sites, while cell‐associated forms (intracellular and cell membrane‐bound) are locally retained. Secreted and cell/EV membrane‐bound forms are more accessible for recognition by B cells and other antigen presenting cells (APCs) than those expressed inside the cells or EVs (Batista & Harwood, 2009; Mintern et al., 2015). As many viral vaccine antigen candidates are trimeric transmembrane glycoproteins, cell and EV membrane‐bound antigens may present antigens in a more native‐like conformation when including their transmembrane domains (Müller et al., 2020). Given the potential to present antigen in different forms, defining their distinctive impact on resultant immunogenicity remains an important focus of research.

EVs (e.g., exosomes) are lipid bilayer‐enclosed structures released by almost all types of cells. Although the exact mechanism underlying EV biogenesis remains elusive, “cargo” loading onto EVs is known to be a selective process. Previous studies have shown that several viral glycoproteins are expressed on the surface of EVs (Mori et al., 2008; Vallhov et al., 2011). Furthermore, earlier studies have shown that the transmembrane (TM) domain of vesicular stomatitis virus (VSV) G protein contains an EV‐targeting signal and that fusion proteins containing VSVG TM are loaded onto the EV surface (Meyer et al., 2017). Whether EV‐targeting can be achieved by other viral glycoprotein TMs and whether the EV‐targeting efficiency would differ by different TMs has yet to be investigated.

In the current study, using GFP as a reporter and model antigen, we compare the EV‐targeting efficiency of TM domains from a range of viral glycoproteins. Further, we investigate whether EV‐bound GFP (EV‐GFP) benefits immune induction over secreted, intracellular and cell membrane‐bound forms. Our results illustrate that different TM domains vary in efficiency of EV incorporation, and that EV presented GFP enhances both humoral and cell‐mediated responses relative to secreted, intracellular and cell membrane‐bound forms in mice. Collectively, our study here indicates that presentation of antigen on EVs using viral TM domains enhances antigen immunogenicity. These findings offer new insights that underlie the high potential of using EVs in nucleic acid vaccine design and development.

## 2 RESULTS

### 2.1 Viral TMs promote the GFP expression on EVs

**FIGURE 1:**
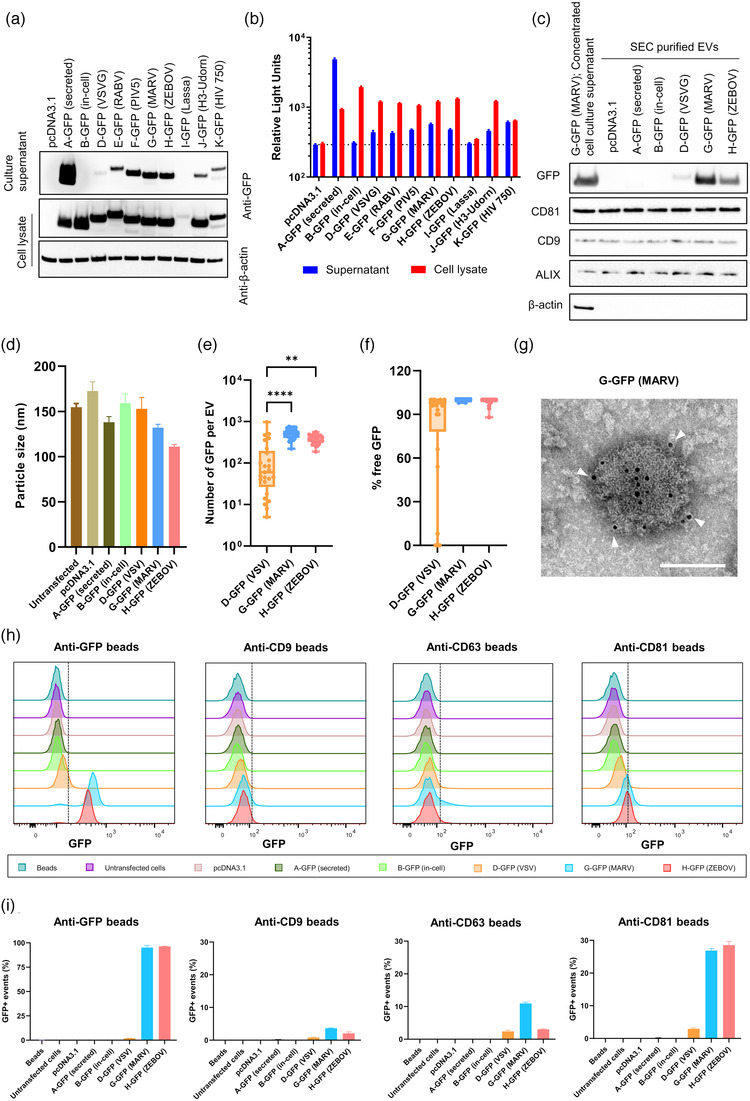
Viral TMs promote the GFP expression on EV surface. HEK293T/17 cells were transfected with different GFP plasmids for 24 h and then (a) GFP expression in the cell culture supernatant and cell lysate was determined by Western blot. A representative result from three independent experiments is shown. (b) GFP fluorescence in the cell culture supernatant and cell lysate was quantified with a microplate reader. Data shown are mean ± SD of three independent experiments. (c–h) EVs in the cell culture supernatant were purified by SEC and then (c) expression of GFP, CD81, CD9, ALIX and β‐actin was determined by Western blot. (d) The size of purified EVs was measured by NTA. (e and f) The number of GFP per EV was determined by FCS (e) and detection of free GFP upon proteinase K treatment (f) confirms surface localization. ^**^, *p* < 0.01; ^****^, *p* < 0.0001. (g) Transmission electron micrograph of negative stained and immunogold‐labeled EVs. G‐GFP EVs were immuno‐labelled with an anti‐GFP primary antibody and a gold nanoparticle (6 nm)‐conjugated IgG. White arrow heads indicate gold nanoparticles. Scale bars represent 100 nm. (h) Purified EVs (10 μg) were pulled down with anti‐GFP, CD9, CD63 or CD81 beads and the GFP signal in the beads was then evaluated by flow cytometry. (h) One representative result is shown. (i) Data shown are mean ± SD of three independent experiments

To test if TMs from viral glycoproteins could enhance the incorporation of antigens on the surface of EVs, we adopted superfolder GFP (sfGFP) (Pédelacq et al., 2006) as a model antigen and constructed a range of plasmids encoding a secreted version of sfGFP fused with TMs and cytoplasmic tails (CTs) from various viral membrane glycoproteins (Figure S1 and Table S1). A secreted soluble GFP control (A‐GFP) and an in‐cell GFP control (B‐GFP) were also designed (Figure S1 and Table S1). All constructs were first transiently expressed in HEK293T/17 cells and the GFP signal in both the cell culture supernatant and cell lysate were determined by Western blot and GFP fluorescence intensity quantification. As expected, A‐GFP, the secreted version, showed a very high level of expression in cell culture supernatant, while B‐GFP, the in‐cell GFP demonstrated dominant expression in cell lysate in Western blot (Figure 1a). The viral TM‐containing GFP constructs, with the exception of I‐GFP, showed similar levels of GFP expression in the cell lysate, but displayed differential expression profiles in cell culture supernatant. F‐GFP, G‐GFP and H‐GFP displayed the highest GFP expression in cell culture supernatant, followed by E‐GFP, then J‐GFP and K‐GFP. Lower expression seen for D‐GFP and almost no expression detected for I‐GFP (Figure 1a). Since all the TM‐containing GFP constructs should in theory only produce membrane‐bound GFP, GFP in the cell culture supernatant from these samples may reflect levels of release of EV‐bound GFP (EV‐GFP). Thus, different levels of GFP in the culture supernatant indicated that different viral TMs could target GFP to EVs with varied efficiencies. Of note, I‐GFP showed barely detectable GFP expression in both cell lysate and cell culture supernatant, indicating that fusion of Lassa TM and CT at the C terminal of GFP may have severely affected expression. Quantified GFP fluorescence also showed a comparable pattern to the Western blot data, indicating that fusing TMs and CTs to GFP did not significantly impact the functionality of GFP, or at least the GFP fluorescence detected by a plate reader (Figure 1b).

We then chose two high EV‐GFP producing constructs (G‐GFP and H‐GFP), one low EV‐GFP producing construct (D‐GFP), together with controls (A‐GFP and B‐GFP) for further characterization. To further determine whether GFP released from cells was EV‐associated, EVs were first purified from cell culture supernatants by size‐exclusion chromatography (SEC), and then were characterized by Western blot, nanoparticle‐tracking analysis (NTA), fluorescence correlation spectroscopy (FCS), immuno‐transmission electron microscopy (TEM) and beads‐based flow cytometry (Figures 1c–i and S2–S5). Western blot showed that strong GFP signal was detected in EVs purified from G‐GFP and H‐GFP transfected cells, weak GFP signal was detected in EVs from D‐GFP transfected cells while no GFP was detected in EVs from pcDNA3.1, A‐GFP and B‐GFP cells (Figure 1c). EVs from all samples were positive for exosome markers CD9, CD81 and ALIX and free of contaminant protein β‐actin (Figure 1c). Particle size analysis by NTA showed that EVs from all samples were between 100 to 200 nm, but there were some variations between different samples. In particular, H‐GFP EVs seemed to be slightly smaller in size than the rest (Figures 1d and S3).

To further determine the level of GFP association with EVs and the localization of GFP in EVs, FCS analysis of EVs with or without proteinase K treatment was performed. FCS is a highly sensitive method to obtain hydrodynamic diameters, enzyme kinetics, and number of cargo loading/release or surface functionalization on nanoparticles (Ariotti et al., 2021; Massi et al., 2020; Rigler et al., 1993; Rigler & Meier, 2006). Incubation of EVs with proteinase K is a common method to study localization of proteins in/on EVs (Ariotti et al., 2021; Bonsergent et al., 2021; Charoenviriyakul et al., 2018). The level of GFP association with EVs was first quantified with EVs in PBS. As shown in Figures 1e and S4, the FCS analysis revealed characteristic nanoparticle diffusion times for all three EV samples confirming that GFP is associated with the nanoparticles. Using the molecular brightness of free GFP (CPP) and of the EV samples further allowed calculating that D‐GFP EVs contained an average of 158 GFP molecules per EV, while G‐GFP EVs and H‐GFP EVs, consistent with the Western blot data, had significantly more GFP loaded per particle, with an average number of 521 and 381 GFP molecules per EV, respectively. EVs were then subjected to proteinase K treatment to measure the GFP content on the EV surface. Proteinase K treatment did not alter the total fluorescence intensity (CR) but led to a sharp decrease of fluorescence signal per particle (CPP), accompanied with faster diffusion times (smaller diameter) and a large increase in the number of diffusing particles (N). This indicates that most GFP molecules were present on the EV surface and were cleaved off by proteinase K, yielding a huge number of now freely diffusing GFP (Figure S4). Because FCS only detects particles with fluorescent signal, EVs free of GFP after proteinase K treatment and control EVs without GFP (EVs from untransfected HEK293T/17 cells) cannot be detected. The total percentage of free GFP upon proteinase K treatment of EVs was also calculated. As shown in Figure 1f, a near 100% free GFP was detected in G‐GFP and H‐GFP EVs, indicating GFP was almost uniquely localized on the surface of these EVs. In D‐GFP EVs, although high level of free GFP was detected, the average percentage of free GFP was slightly lower at 79%. This implied that D‐GFP EVs also carried a small portion of GFP inside the EVs.

The presence of GFP on EVs was further analysed by immuno‐transmission electron microscopy (TEM). G‐GFP EVs and native EVs (EVs purified from untransfected HEK293T/17 cells) were immunolabelled with an anti‐GFP antibody and IgG‐conjugated gold nanoparticles, and negatively stained for contrast. The micrographs clearly show a high density of gold nanoparticles labelling the GFP on the surface of G‐GFP EVs, while nanoparticle labelling was completely absent for the control native EVs, further validating the GFP expression on EV surface (Figures 1g and S5). As the TEM imaging was performed on fixed and dehydrated EVs, the morphological and size information from these TEM micrographs may not represent that of the actual EVs. Instead, the NTA data on the size information of the EVs would be more accurate because the EVs were analysed in their native hydrated state (Figures 1d and S3).

To determine the GFP association with different EV subsets, purified EVs were pulled down by magnetic beads coated with either anti‐GFP, anti‐CD9, anti‐CD63 or anti‐CD81 antibodies and the GFP signal on beads was then evaluated by flow cytometry. Consistent with our Western blot results in Figure 1c, GFP signal was only detected in high levels in G‐GFP and H‐GFP and at a low level in D‐GFP (Figure 1h–i). In addition, although GFP was detectable in CD9‐, CD63‐ and CD81‐positive EVs, the EV‐GFP seemed to be predominantly associated with the CD81+ subset (Figure 1h–i). Together, these data confirmed that viral TM‐containing constructs produced EV‐bound GFP.

### 2.2 Subcellular distribution of GFP in transfected cells

**FIGURE 2:**
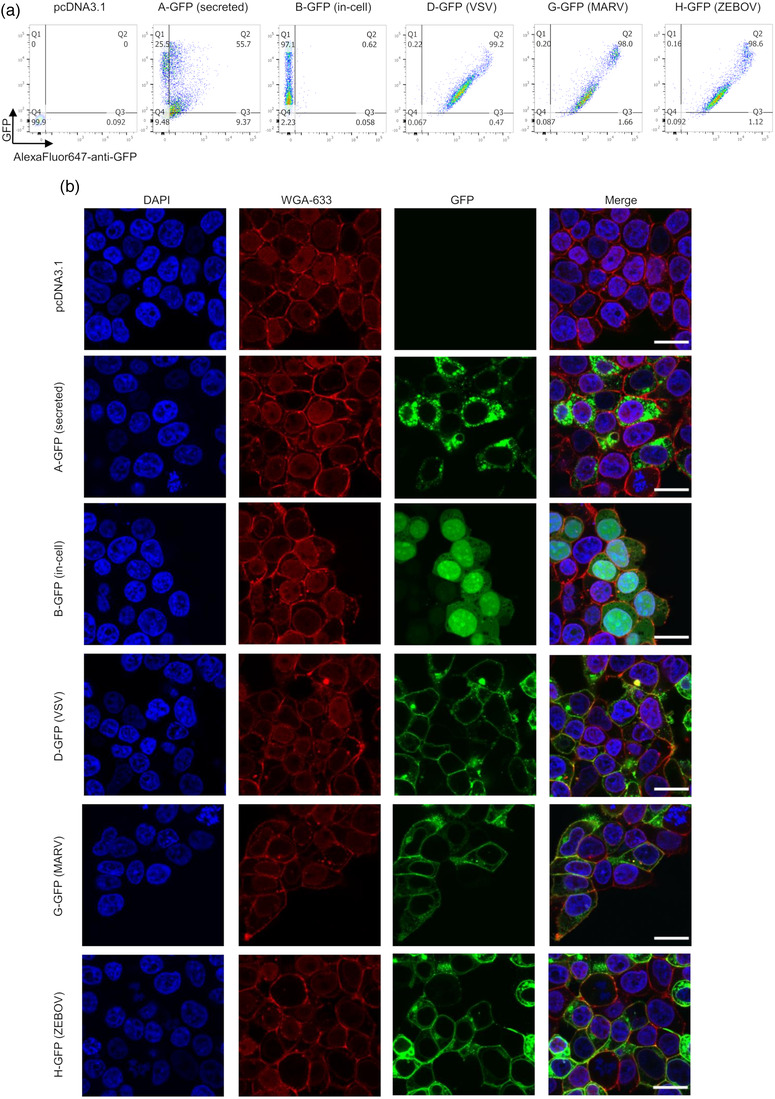
Subcellular distribution of GFP in transfected cells. (a and b) HEK293T/17 cells were transfected with different GFP plasmids for 24 h and then (a) cell surface GFP was stained with anti‐GFP‐AF647 and GFP and AF647 signals were recorded by flow cytometry. A representative result from three independent experiments is shown. (b) cells were first fixed with 4% PFA and then cell membrane and nucleus were stained by WGA633 and DAPI, respectively. Stained cells were imaged by confocal microscopy. One representative result from three independent experiments is shown. Scale bar: 10 μm

When a vaccine is launched as DNA or RNA, the antigen is expressed in transduced cells and then is either secreted, remains inside the cells and/or is expressed on the cell membrane surface. We next tested the subcellular GFP distribution expressed by different GFP constructs. HEK293T/17 cells were first transfected with different GFP constructs, and cell surface GFP was stained with the AF647‐anti‐GFP antibody. Flow cytometry detection of both whole cell‐derived GFP fluorescence (*y* axis) and AF647 signal (cell surface GFP; *x* axis) revealed distinctive GFP expression patterns by different constructs (Figures 2a and S6). A‐GFP, the secreted soluble GFP, showed both intracellular and cell surface expression, with a stronger signal inside the cells than on the surface. B‐GFP, the in‐cell GFP, expectedly showed full intracellular expression. The viral TM‐containing constructs (D/G/H‐GFP) showed very high correlations between GFP fluorescence and AF647 signal, indicating the localization of GFP on the cell membrane (Figures 2a and S6). Similar results were observed in fluorescent microscopy (Figure 2b). Of note, in addition to the predominant GFP signal on the cell membrane surface, in cells transfected with viral TM‐containing constructs, there was also noticeable GFP signal associated with the intracellular membrane structures, especially apparent for G‐GFP and H‐GFP, indicating pathways of GFP‐EV genesis (Figure 2b).

Taken together, our data above indicate that different GFP constructs produce distinctive forms of GFP (Table. S2). In detail, A‐GFP produces soluble, intracellular and cell surface GFP; B‐GFP produces mainly intracellular GFP; D‐GFP produces cell surface, intracellular GFP as well as a small amount of EV‐GFP; and both G‐GFP and H‐GFP produce the same types of GFP as D‐GFP, but generate higher levels of EV‐GFP.

### 2.3 EV‐GFP enhances antigen‐specific humoral and cell‐mediated responses

**FIGURE 3:**
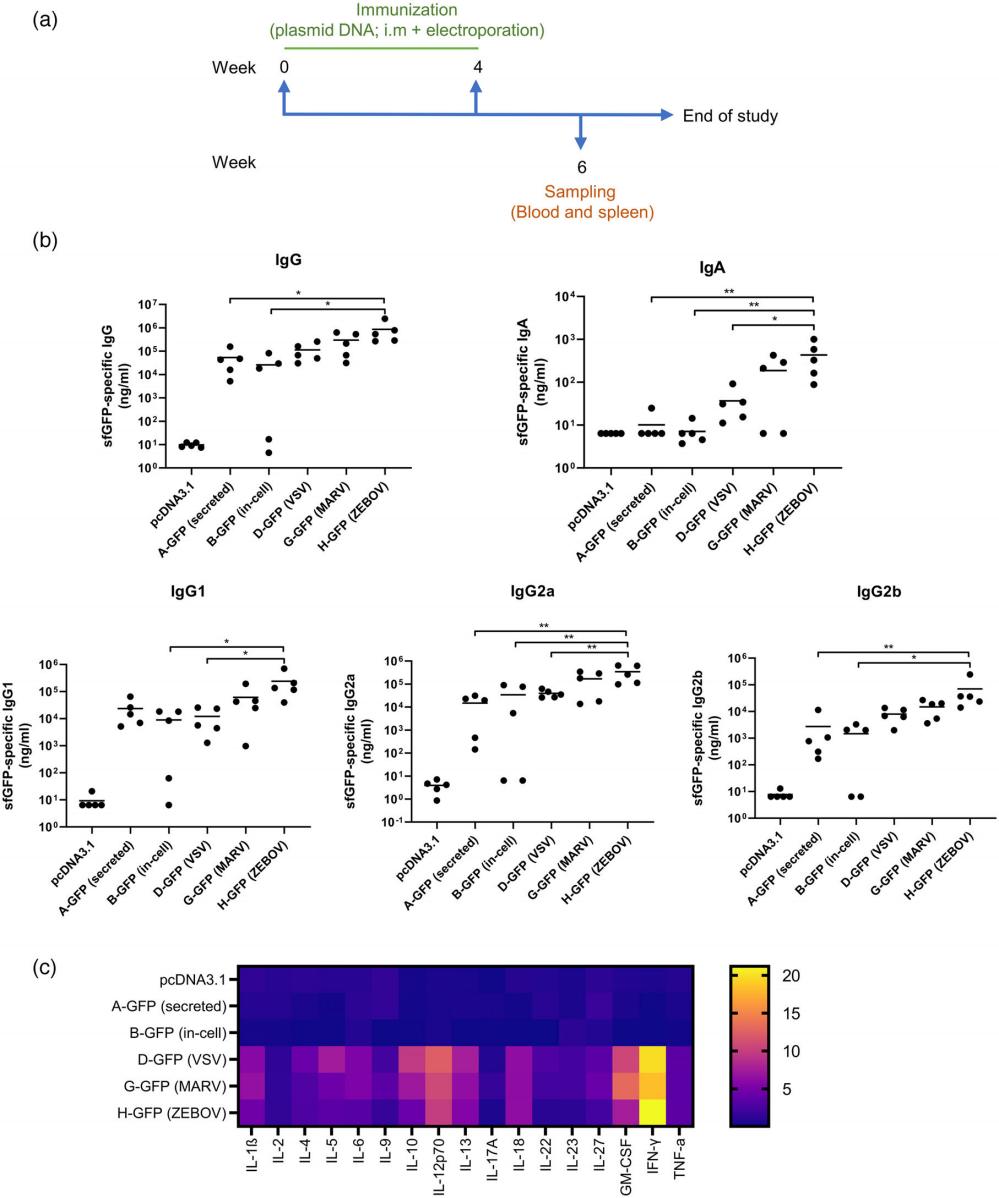
Immunization of EV‐GFP‐producing plasmids enhances antigen‐specific humoral and cellular responses. BALB/c mice were injected with different GFP plasmids (10 μg DNA in 50 μl PBS) twice at 4‐week intervals. (a) Immunization schedule is shown. (b) Two weeks after the second injection, blood was collected and GFP‐specific IgG, IgA, IgG1, IgG2a and IgG2b were quantified by ELISA (*n* = 5). ^*^, *p* < 0.05; ^**^, *p* < 0.01. (c) Two weeks after the second injection, mice were sacrificed and splenocytes were prepared and restimulated with purified GFP for 7 days. Following stimulation, cytokines released by the splenocytes were quantified by a Th1/Th2/Th9/Th17/Th22/Treg Cytokine 17‐Plex Mouse ProcartaPlex™ Panel (*n* = 5)

First, we investigated whether the expression of GFP plasmids in vivo would also generate GFP EVs. Mice were injected intramuscularly (i.m.) with different GFP plasmids and 24 h later, injection site muscle was harvested, and cells were isolated and GFP+ cells were quantified. As shown in Figures S7A and B and S11, GFP expression was successfully detected in cells isolated from the injection sites and similar levels of expression were detected among different groups. Cells isolated from the injection sites were also cultured in vitro and EVs were purified from culture media and GFP signal was evaluated by anti‐GFP beads‐based flow cytometry. Our data confirmed that GFP EVs were detected in muscles injected with G‐GFP but not A‐GFP (Figure S7C). We next investigated the differential impact of the various forms of GFP presentation on immune induction in a BALB/c mouse model. Mice were first i.m. injected twice (0 and 4 weeks) with different GFP plasmids, blood samples were collected 2 weeks after the final injection, and GFP‐specific antibody titers were measured (Figure 3a). All GFP plasmids induced GFP‐specific IgG and IgA responses (Figure 3b). The antibody responses in A‐GFP and B‐GFP injected animals were comparable, while both IgG and IgA titers were elevated in animals that received the EV‐GFP‐producing plasmids (D/G/H‐GFP). In particular, the IgG titer in H‐GFP group was significantly higher than that in A‐GFP and B‐GFP, showing an increase of 16‐fold (*p* = 0.0333) and 33‐fold (*p* = 0.0260), respectively. An even more profound enhancement by EV‐GFP‐producing plasmids was observed in GFP‐specific IgA. A‐GFP and B‐GFP groups had close‐to‐background IgA level while considerably higher level of IgA was detected in D/G/H‐GFP groups and statistical significance was reached in H‐GFP group compared to A‐GFP (43‐fold increase, *p* = 0.0069), B‐GFP (61‐fold increase, *p* = 0.0065) and D‐GFP (12‐fold increase, *p* = 0.0125). GFP‐specific IgG subtypes (IgG1, IgG2a and IgG2b) were also quantified in all groups. Similar to the GFP‐specific IgG response data, A‐GFP and B‐GFP induced comparable responses across all three immunoglobulin subtypes. D‐GFP did not show apparent increase in IgG1 and IgG2a, but a slight increase in IgG2b compared to A‐GFP and B‐GFP. Both G‐GFP and H‐GFP induced stronger responses across the three immunoglobulin subtypes over the two control groups, but statistical significance was only detected in H‐GFP.

The cell‐mediated response was also investigated by quantification of Th‐associated cytokine production from specific antigen‐restimulated splenocytes. Two weeks after the final injection, mice were sacrificed and splenocytes were prepared and restimulated in vitro with purified GFP and Th1/Th2/Th9/Th17/Th22/Treg‐associated cytokines in the culture medium were quantified. No apparent increase above background (pcDNA3.1 group) was detected in A‐GFP and B‐GFP immunized mice for all the cytokines measured (Figures 3c and S8). By contrast, many of the 17 measured cytokines were considerably increased in mice that received the EV‐GFP‐producing plasmids (D‐GFP, G‐GFP and H‐GFP). In particular, IL‐12p70, GM‐CSF and IFN‐γ showed more than 10‐fold increase in all three EV‐GFP‐producing plasmids groups. Interestingly, although H‐GFP showed the highest enhancement in GFP‐specific antibody responses, it did not show greater enhancement than D‐GFP and G‐GFP in the production of Th‐related cytokines.

**FIGURE 4:**
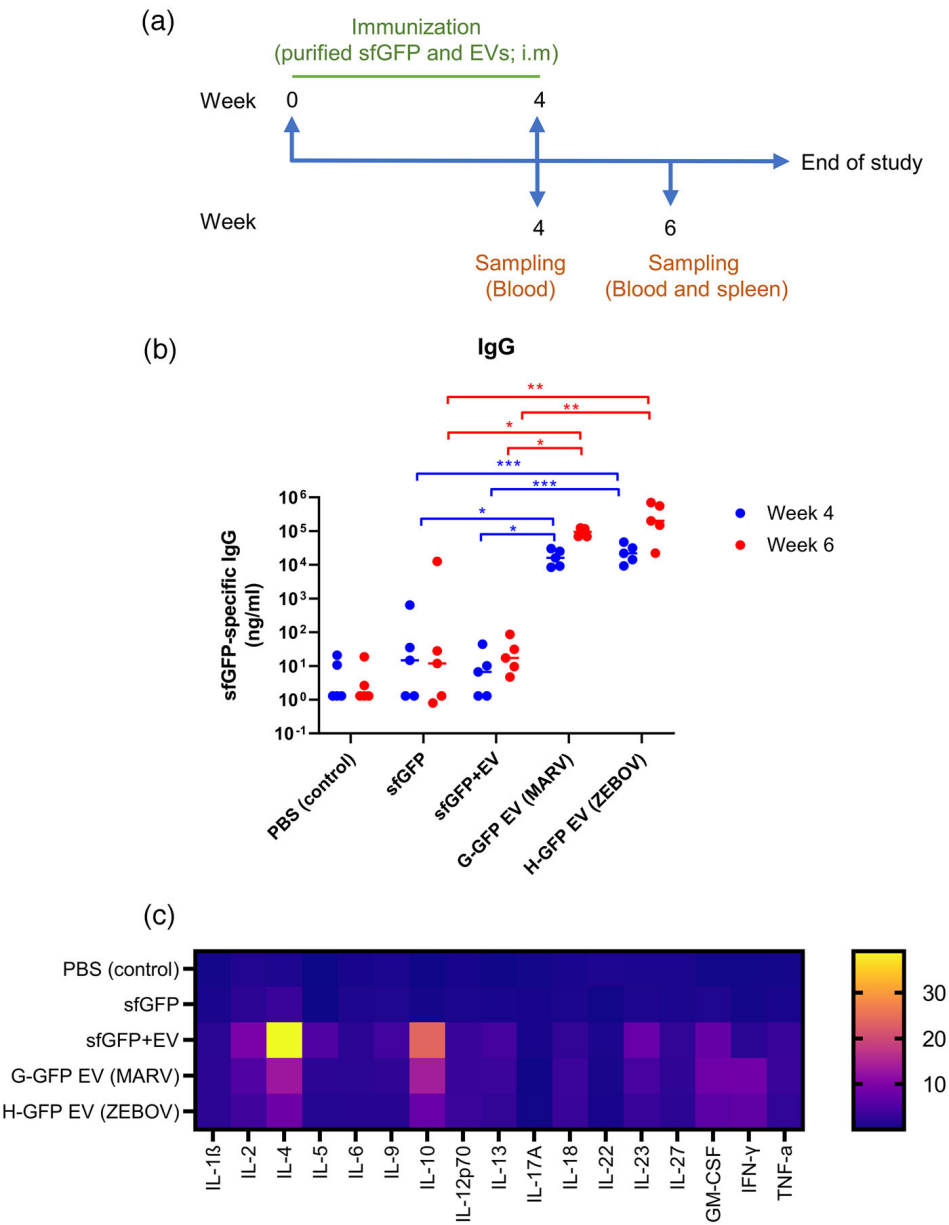
EV‐GFP enhances antigen‐specific humoral and cell‐mediated responses. Mice were injected twice with 2 μg purified soluble GFP (sfGFP), 2 μg sfGFP + 6 μg purified native EV, 8 μg G‐GFP EV, or 8 μg H‐GFP EV at 4‐week intervals. (a) Immunization schedule is shown. (b) Blood was collected at week 4 and 6 and GFP‐specific IgG titers were quantified by ELISA (*n* = 5). ^*^, *p* < 0.05; ^**^, *p* < 0.01; ^***^, *p* < 0.001. (c) Two weeks after the second injection, mice were sacrificed and splenocytes were prepared and restimulated with purified GFP for 7 days. Following stimulation, cytokines released by the splenocytes were quantified by a Th1/Th2/Th9/Th17/Th22/Treg Cytokine 17‐Plex Mouse ProcartaPlex™ Panel (*n* = 5)

To further confirm that EV‐GFP possesses the immune enhancement capability, another mouse study was conducted. Mice were immunized twice, 4‐weeks apart, with either purified GFP (sfGFP), sfGFP + native EVs (EVs purified from untransfected HEK293T/17 cells), G‐GFP EVs or H‐GFP EVs and then GFP‐specific antibody and cell‐mediated responses were measured (Figure 4a). Our data showed that G‐GFP and H‐GFP EVs induced significantly higher GFP‐specific IgG response than sfGFP alone or a physical mix of sfGFP + EVs (Figure 4b). Similarly, Th1/Th2/Th9/Th17/Th22/Treg‐associated cytokines were elevated in G‐GFP and H‐GFP EVs groups compared to the sfGFP group (Figures 4c and S9). Interestingly, the sfGFP + EVs group showed a comparable increase in the production of GFP‐specific Th‐related cytokines, implying that the inclusion of native EVs has immunomodulatory effect on antigen‐specific cellular response (Figures 4c and S9). Taken together, our data indicate viral TM‐containing GFP plasmids can produce EV‐GFP in vivo and that EV‐GFP induces better humoral and cellular immune responses.

### 2.4 EV‐GFP is more efficiently taken up by splenocytes

**FIGURE 5:**
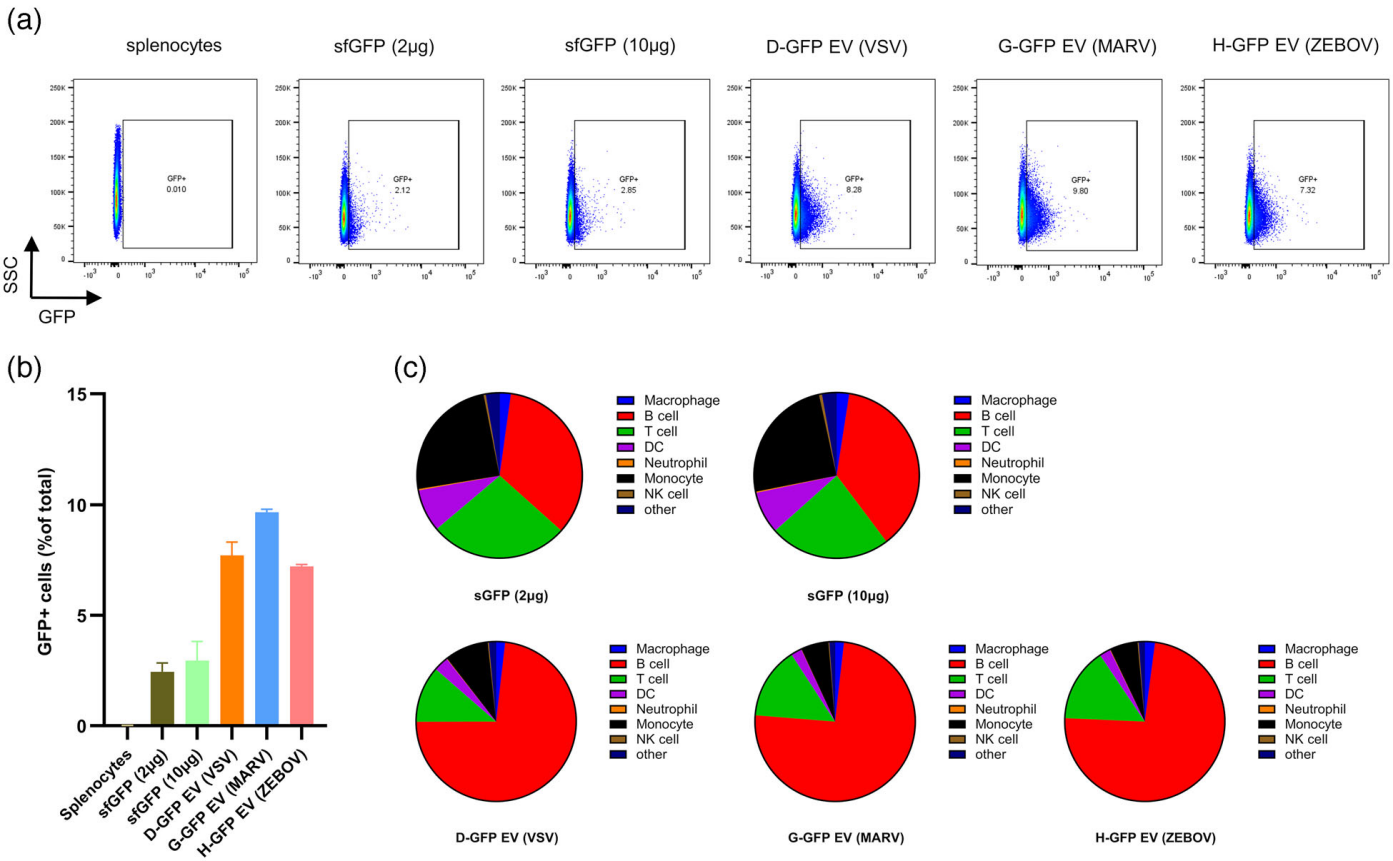
EV‐GFP is more efficiently taken up by splenocytes. (a–c) Splenocytes were isolated from naïve BALB/c mice and incubated with either purified soluble sfGFP (2 and 10 μg), purified D‐GFP EV (10 μg), G‐GFP EV (10 μg) or H‐GFP EV (10 μg) for 1 h, and then (a) GFP+ cells were quantified by flow cytometry and (b) the percentage of GFP + cells was calculated. Data shown are mean ± SD of three independent experiments. (c) Cells were stained with the following cell subtype markers B cell (CD19‐BV605), T cell (CD3e‐BV711), DC (CD11c‐BV785), NK cell (CD49b‐APC/Cy7), macrophage (F4/80‐BV421), monocyte (CD11b‐PE) and neutrophil (Ly6G‐BV650), and GFP+ cell subtypes were analysed. Data shown are mean of three independent experiments

Antigen presentation is an essential process for the induction of an adaptive immune response. During this process, antigen is taken up, processed, and presented to T cells by APCs (Lanzavecchia, 1996). Effective immune responses are typically driven by professional APCs, including macrophages, B cells and dendritic cells, etc. although a wide range of cell types can act as APCs under certain circumstances (Weaver & Unanue, 1990). Having observed that EV‐GFP could induce stronger humoral and cell‐mediated responses than soluble/intracellular/cell surface GFP, we explored whether different forms of GFP could affect the uptake by APCs. To this end, splenocytes isolated from naïve mice were treated with purified soluble sfGFP (2 and 10 μg), purified D‐GFP (10 μg), G‐GFP (10 μg) or H‐GFP EVs (10 μg purified G‐GFP EVs contained roughly 1.6 μg GFP and 10 μg H‐GFP EVs contained approximately 1.1 μg GFP. Figure S10). GFP uptake by splenocytes was quantified by flow cytometry. Interestingly, there were considerably more GFP+ cells on exposure to EV‐GFP (D/G/H‐GFP) than sfGFP, despite the dose of sfGFP used (Figures 5a and b and S11). Cell subtypes analysis indicated that EV‐GFP was predominantly taken up by B cells while soluble GFP was taken up by more diverse cell subclasses (Figured 5c, S11 and 12). Taken together, our data indicate that EV‐GFP can be taken up more efficiently than soluble GFP and that EV‐GFP uptake is B cell preferential.

## 3 DISCUSSION

Antigen optimization to improve immunogenicity remains an important focus of vaccine development. Many studies have concentrated on modifications that aim to preserve the native antigen structure, remove or mask non‐neutralizing epitopes and/or focus responses on neutralizing epitopes (Aldon et al., 2018; Huang et al., 2012; Kovacs et al., 2014; Krebs et al., 2014). More recently, the potential of EVs as a vehicle for enhancing vaccine immunogenicity has become an area for active research. Several EV‐based vaccines have successfully induced T cell responses against tumour antigens (Altieri et al., 2004; Hartman et al., 2011) and both B cell and T cell responses against viral antigens (Kuate et al., 2007; Wang et al., 2017). However, the advantages of EV‐bound antigen over other antigen presentations remain an open question. In this current study, we have shown that the inclusion of TM domains from viral glycoproteins represents a simple but effective way to target protein antigens to EV. Consistent with earlier findings, our study confirms that EV‐bound antigen induces antigen‐specific B and T cell responses (Kuate et al., 2007; Wang et al., 2017). More importantly, our study has further shown that the humoral and in particular cell‐mediated responses induced by EV antigens are significantly stronger than those induced by secreted soluble, intracellular and cell surface forms. The EV‐antigen induced immune enhancement may be a result of preferential B cell‐ uptake of EV‐antigen forms. These findings demonstrate the potential value of EVs in vaccine design.

One of the big challenges in EV‐based vaccine development is antigen incorporation into/onto EVs. Currently, there are two main approaches for antigen incorporation. One is to produce and purify EVs from antigen‐expressing cells while the other is to attach the antigen to an EV‐targeting signal. The first approach is a simple process, but its utilization is limited to antigens with inherent EV‐targeting signal where passive EV loading process may not be very efficient (Altieri et al., 2004; Yin et al., 2013). The second approach can actively load a wide range of antigens onto EVs and many techniques have been investigated for this approach (Donoso‐Quezada et al., 2020). Among the various techniques, plasma membrane anchors have been shown to increase protein loading into EVs, but the loading efficiency varies dramatically with different anchors and proteins and the various combinations and proteins can only be loaded inside the EVs (Shen et al., 2011). Similar issues are also present when using ESCRT‐interacting peptide tags and EV‐targeting viral proteins (Anticoli et al., 2018; Cheng & Schorey, 2016; Villarroya‐Beltri et al., 2014). Decoration of proteins on the EV surface through conjugation has overcome these problems and has been proven to be highly effective (Hosseini Shamili et al., 2019). However, the conjugation process is rather complex and therefore may not be cost‐effective. The use of TMs from viral glycoproteins as EV‐targeting signals has proven to be a simple but efficient technique to load antigens both on the EV surface as well as inside the EVs (Kuate et al., 2007; Meyer et al., 2017). In addition, unlike the protein‐EV conjugation technique, it can be encoded in DNA/RNA vaccines, enabling the production of antigen‐loaded EVs in vivo. Of note, limitations remain for the viral TM‐derived EV‐targeting signal approach. As for other fusion proteins, fusing viral TMs onto antigens may potentially affect the expression and/or conformation and function of the fusion products. In our study, we have noticed that the addition of Lassa TM to GFP led to almost no expression of the fusion protein. In addition, the GFP fluorescence intensity data (Figure 1b) was not strictly correlated with the GFP expression level (Figure 1a). For instance, K‐GFP showed considerably lower GFP expression in the culture supernatant than F/G/H‐GFP by Western blot, but showed comparable fluorescence intensities to the other three. Both observations indicate that the expression and conformation of GFP are to some extent affected by the TM tagging.

Our study has shown that EV‐bound antigen was preferentially taken up by B cells, unlike the soluble version of GFP that had a more diverse cell subtype profile. While cell‐specific EV uptake has not been extensively studied, our findings are consistent with a previous study showing that mantle cell lymphoma‐derived exosomes were selectively internalized by B cells (Hazan‐Halevy et al., 2015). Besides their role in adaptive humoral response, B cells are also important APCs. Of note, even though B cells can recognize antigen in various forms, they show a predominant response to membrane‐bound antigens (Batista & Harwood, 2009). This may explain the preferential uptake of EV‐bound antigens by B cells compared to soluble antigens. Furthermore, although B cells are not the only type of APCs, they provide essential antigen presentation capacity above that provided by DCs, which is crucial for T cell expansion and generation of memory and effector T cells (Crawford et al., 2006). Of note, the EV uptake assays in our current study and the previous study were both done in vitro, therefore, further investigation is required to confirm the mechanism of action in vivo (Hazan‐Halevy et al., 2015). We were unable to detect any GFP signal in the draining lymph nodes and spleen in immunized mice (Data not shown). We suspect that the GFP EV release and uptake by cells in vivo is a continuous process that occurs at a very low‐frequency and may be rapidly degraded. Although this is enough to induce an immune response, it is too low to be detected by current techniques such as in vivo imaging and flow cytometry. Further investigation is still required to decipher the mechanism of EV‐bound antigen enhanced immune response, but it is likely that efficient uptake of EV‐antigen by B cells improves the T cell response toward the antigen, which in turn leads to enhanced antigen‐specific humoral and cell‐mediated responses. It would also be interesting to investigate whether EVs with different viral TMs have an impact on EV biodistribution and half‐life, however this is likely to require a more sensitive marker and falls beyond the scope of this current study.

A 24 h time point was selected for most of our in vitro studies, because GFP shedding from EV/cell surface was detected beyond this time point (Figure S13). Ectodomain shedding is a type of highly regulated post‐translation modification, with its underlying mechanisms largely unclear (Lichtenthaler & Meinl, 2020). Although the exact mechanism underlying GFP shedding from EV and/or cell surface remains elusive, it is very likely that, based on our data, the shedding was a result of cell stress accumulation under the artificial in vitro culture condition. When cells were cultured for extended time periods, GFP shedding from E‐GFP (RABV) sample increased along with the cell culture time (Figure S13). Meanwhile, intracellular GFP release into cell culture media from B‐GFP (in‐cell) sample also increased when cell culture time was extended, indicating that cells were stressed, and that cell death was increased (Figure S13). Since the cell stress from the artificial in vitro culture condition does not happen in vivo, it is possible that such GFP shedding we observed in cell culture may not occur in vivo. According to previous studies, the protein ectodomain shedding rate is only about 2%, therefore, GFP shedding in vivo, if it happens, would be much less profound (Arribas & Borroto, 2002; Hayashida et al., 2010). Under the scenario of in vivo GFP shedding, shed GFP would be just as immunogenic as the soluble GFP. Since our data showed that soluble GFP or soluble GFP physically mixed with native EVs did not induce an immune response as strong as the GFP‐EVs, it is safe to conclude that presentation of GFP on EV surface enhances GFP immunogenicity.

Taken together, our present study shows that efficient EV‐loading of proteins can be achieved by a simple inclusion of TMs from viral glycoproteins, that the EV‐bound antigen form is preferentially taken up by B cells and induces enhanced humoral and cell‐mediated responses in mice. The findings of this study provide valuable information in the application of EV in vaccine development and a novel approach for antigen design.

## 4 MATERIALS AND METHODS

### 4.1 Cells

The HEK293T/17 cell line was purchased from LGC standards and cultured in DMEM supplemented with 10% FBS (Gibco, Thermo Fisher Scientific), L‐glutamine (Gibco, Thermo Fisher Scientific) and Penicillin–Streptomycin (Merck).

Primary murine lymphocytes from the spleen (splenocytes) and draining lymph nodes were prepared using Lymphoprep™ density gradient (StemCell) with SepMate tubes (StemCell), according to the manufacturer's instruction. In brief, spleens and draining lymph nodes were freshly isolated in serum‐free DMEM and pushed through 70 μm cell strainers (BD Biosciences) to generate single cell suspensions. The cell suspensions were then layered onto Lymphoprep™ density gradient in the 15 ml SepMate tubes and centrifuged at 800 × *g* for 30 min in swing buckets without breaks. Following centrifugation, the separated lymphocytes were poured into new 15 ml Falcon tubes and washed twice with serum‐free DMEM. After the final wash, cells were resuspended and cultured in DMEM supplemented with 10% FBS (Gibco, Thermo Fisher Scientific), L‐glutamine (Gibco, Thermo Fisher Scientific) and Penicillin–Streptomycin (Merck).

Primary murine muscle cells were isolated as previously described with modifications (Blakney et al., 2021). In brief, mouse muscle was freshly isolated and digested in 3 ml of DMEM supplemented with 1 mg/ml collagenase P (Merck) and 5 mg/ml Dispase II (Merck) for 45 min at 37°C on an orbital shaker. After digestion, single cell suspension was generated by pushing the digests through a 70 μm cell strainer (BD Biosciences). Cells were then washed twice with serum‐free DMEM, either resuspended in PBS and used directly for flow cytometry or resuspended in skeletal muscle cell growth medium for culture.

### 4.2 Plasmids

Superfolder GFP (sfGFP) was used as a model antigen in the current study. To generate a secreted soluble sfGFP construct (A‐GFP), the secretion signal sequence from rat follicle stimulating hormone beta‐subunit (GenBank Accession number: M36804.1) was added to the N terminus of sfGFP (Roh et al., 2013). sfGFP without a secretion signal (B‐GFP) was also constructed as an in‐cell antigen control. To generate constructs expressing sfGFP fused to viral transmembrane (TM) and cytoplasmic domains (CT), the A‐GFP sequence was followed by a (G_4_S)_2_ linker and various viral TMs and CTs. The following viral glycoprotein TMs and CTs were used in the current study: vesicular stomatitis virus glycoprotein (VSV‐G; GenBank Accession number: AKE31777.1; D‐GFP), rabies glycoprotein (RABV‐G; GenBank Accession number: AEV43289.1; E‐GFP), parainfluenza virus 5 fusion glycoprotein (PIV5‐F; GenBank Accession number: AAC95515.1; F‐GFP), Marburgvirus glycoprotein (MARV‐G; GenBank Accession number: AFV31349.1; G‐GFP), Zaire ebolavirus glycoprotein (ZEBOV‐G; GenBank Accession number: AKI84258.1; H‐GFP), Lassa mammarenavirus glycoprotein (Lassa‐G; GenBank Accession number: AIT17828.1; I‐GFP), influenza virus A hemagglutinin (HA; GenBank Accession number: AFY97264.1; J‐GFP) and Human immunodeficiency virus 1 envelope glycoprotein (HIV‐1‐Env; GenBank Accession number: ASW20076.1; K‐GFP). A His‐tagged version of the A‐GFP plasmid (A‐GFP‐His) was also constructed by adding an 8*His tag to the C terminal of A‐GFP to enable protein purification by Nickel‐affinity chromatography. All coding sequences were synthesized as strings by GeneArt (Thermo Fisher Scientific) and assembled into pcDNA3.1 using NEB HiFi assembly (New England BioLabs), according to the manufacturer's instructions. A design of the fusion constructs is shown in Figure S1 and a full list of the constructs is shown in Table S1.

### 4.3 Transfection and GFP expression in vitro

All in vitro transfections were done in HEK293T/17 cells using PEI MAX® (Polysciences), according to the manufacturer's instructions. In brief, HEK293T/17 cells preseeded in 6‐well plates or T175 flasks were transfected with different GFP plasmids using PEI MAX at DNA to PEI = 1:3 ratio in serum‐free DMEM. Four‐to‐six hours post transfection, transfection medium was removed, and cells were cultured in complete DMEM (Sigma‐Aldrich; Merck) or serum‐free Freestyle293 medium (for EV production; Thermo Fisher Scientific). After 24 h, cell culture supernatants and cells were separately collected. Culture supernatants were filtrated through a 0.8 μm membrane to remove cell debris. Cells were lysed with Pierce IP/Lysis buffer (Thermo Fisher Scientific) supplemented with protease inhibitor cocktail (Roche) and insoluble was removed by centrifugation (10,000 × *g*, 10 min at 4°C).

### 4.4 EV purification

EVs produced by HEK293T/17 cells transfected with different GFP plasmids were purified by size exclusion chromatography (SEC) using qEVoriginal/70 nm or qEV10/70 nm (IZON). Cleared conditioned cell culture medium was first concentrated with 100 kD Amicon ultrafiltration tubes (Merck Millipore) and filtrated through a 0.8 μm membrane, and then loaded onto a PBS‐equilibrated SEC column. For EV separation efficiency analysis, qEVoriginal/70 nm column was used, and 20 fractions of 0.5 ml were collected. For other purposes, qEV10/70 nm column was used and only the EV fractions were collected and combined. The collected fractions were then concentrated by 100 kD Amicon ultrafiltration tubes, aliquoted and either directly used for downstream analyses or stored at −80°C.

### 4.5 Nanoparticle‐tracking analysis (NTA)

Freshly purified EVs were characterized by NTA with a Nanosight LM10 machine, according to the manufacturer's instructions (Malvern Instruments). For each sample, three videos of 60 s each were recorded. Data were analysed by NTA software 3.0 and particle average size, distribution and concentration were calculated.

### 4.6 GFP fluorescence intensity measurement

GFP fluorescence intensity in cell culture supernatants and cell lysates was quantified with a FLUOstar Omega microplate reader (BMG LABTECH), according to the manufacturer's instructions. In brief, samples were transferred into a clear bottom black plate and fluorescent signal were recorded using an excitation filter of 485 nm and an emission filter of 520 nm.

### 4.7 Recombinant GFP production and purification

GFP was produced and purified as previously described with modifications (Fu et al., 2019). In brief, HEK293T/17 cells were first transfected with A‐GFP‐His using PEI MAX® (Polysciences) for 4–6 h, and then cells were switched to the serum‐free Freestyle293 medium (Thermo Fisher Scientific) and cultured for 3 days. Conditioned medium was then harvested and filtrated to remove cell debris, and GFP was purified with a HisTrap HP column (Cytiva) on a Bio‐Rad NGC liquid chromatography system. Purified GFP was then washed with PBS and concentrated using the 10 kD Amicon ultrafiltration tubes (Millipore, Merck). The quality of purified GFP was finally determined by SDS‐PAGE and Western blot.

### 4.8 Western blot

The expression of GFP and EV markers were determined by Western blot, as previously described with modifications (Hu et al., 2020). In brief, cell culture supernatant, cell lysate or purified EVs were first mixed with Bolt™ LDS Sample Buffer (Thermo Fisher Scientific). Apart from the detection of CD9 and CD81, reducing reagent (Thermo Fisher Scientific) was also added into the samples. The samples were then heated at 70°C for 10 min and separated by a Bolt™ Bis‐Tris Gel (Thermo Fisher Scientific). Subsequently, the proteins were transferred onto a PVDF membrane using the Trans–Blot Turbo Transfer System (Bio‐Rad). After blocking in 5% nonfat milk for 1 h at room temperature, the membrane was sequentially incubated with primary antibodies overnight at 4°C and secondary antibodies for 1 h at room temperature, respectively. Following antibody incubation, the membrane was extensively washed with PBST and the immuno‐bands were visualized using Immobilon Crescendo Western HRP substrate (Merck Millipore) with a Celvin S chemiluminescence Imager (Biostep). The following primary antibodies were used: rabbit anti‐GFP (ab6556; Abcam), mouse antihuman β‐actin (sc‐47778; Santa Cruz), rabbit antihuman CD9 (ab263019; Abcam), mouse antihuman CD81 (MAB4615; R&D systems), mouse antihuman ALIX (2171; Cell Signaling Technology). The following HRP‐conjugated secondary antibodies were used: mouse antirabbit IgG‐HRP (sc‐2357; Santa Cruz) and m‐IgGκ BP‐HRP (sc‐516102; Santa Cruz).

### 4.9 Fluorescence correlation spectroscopy (FCS)

Samples of purified sfGFP (300 μg/ml) or EV samples (1 × 10^11^ particles/ml) were preincubated at 37°C in PBS (6.5 h) or PBS containing 50 μg/ml proteinase K (NEB, P8107) in PBS (5.5 h). FCS measurements were then recorded using a commercial LSM 880 (Carl Zeiss, Jena, Germany). The incubation chamber of the FCS instrument was kept at a constant temperature of 37°C. An Ar+ laser was used for 488 nm excitation, whilst selecting the appropriate filter sets to detect the fluorescence signal. Fluorescence was measured through a 40× C‐Apochromat water immersion objective (NA = 1.2). The sample volume was 5 μl, which was pipetted into an ibidi 8‐well glass bottom chamber and the beam was focused 200 μm above the bottom glass plate. An Oregon Green 488 (OG488) solution in PBS was employed as a standard to calibrate the beam (D = 5.49 × 10^–6^ cm^2^/s at 37°C, using D = 4.1 × 10^–6^ cm^2^/s at 25°C) (Kapusta, 2010). Intensity traces of 25 × 5 or 10 s were recorded for each sample. The intensity fluctuations were then autocorrelated by ZEN software (Carl Zeiss, Jena, Germany) and analysed using PyCorrfit program 1.1.6 (Müller et al., 2014). For graphs showing autocorrelation curves, the average curves across the whole measurement time are shown, whilst the other plots contain the total of 25 points from all the individual autocorrelation curves. One component fits ($G_{1\mathrm{comp}}\left(\tau \right)$) were used to obtain hydrodynamic diameters (D_h_) and number of sfGFP per particle. To determine the percentage of free sfGFP upon proteinase K treatment, measurements of free sfGFP (3 μg/ml in PBS) were first analysed using one component fits ($G_{1\mathrm{comp}}\left(\tau \right)$) to yield the diffusion time ($\tau_{1}$) and brightness for one sfGFP protein (CPP1 in kHz). Subsequently, two component fits ($G_{2\mathrm{comp}}\left(\tau \right)$) with one component fixed to free sfGFP diffusion time ($\tau_{1}$) were used to analyze EV measurements after treated with proteinase K. Due to the nonproportional contribution of slow diffusing and bright particles (here, particles with sfGFP), (Tcherniak et al., 2009) the fraction of free sfGFP (F_1_) from the fit had to be adjusted with the accompanying decrease in the number of sfGFP per particle (Massi et al., 2020). A triplet fraction with a triplet time fixed between 1 and 10 μs was included for all measurements.

$$G_{1\mathrm{comp}}\left(\tau \right)=\left(1+\frac{T}{1-T}e^{\frac{-\tau }{\tau_{\mathrm{trip}}}}\right)\;*\frac{1}{N*\left(1+\frac{\tau }{\tau_{D}}\right)*\sqrt{1+\frac{\tau }{SP^{2}\tau_{D}}}}$$

$$G_{2\mathrm{comp}}\left(\tau \right)=\left(1+\frac{T}{1-T}e^{\frac{-\tau }{\tau_{\mathrm{trip}}}}\right)\;*\frac{1}{N}*\left[\frac{F_{1}}{\left(1+\frac{\tau }{\tau_{1}}\right)*\sqrt{1+\frac{\tau }{SP^{2}\tau_{1}}}}+\frac{1-F_{1}}{\left(1+\frac{\tau }{\tau_{2}}\right)*\sqrt{1+\frac{\tau }{SP^{2}\tau_{2}}}}\right]$$

$$\text{CPP}\;\text{total}\;=\;F1*\mathrm{CPP}1+\left(1-F1\right)*\mathrm{CPP}2$$

NumberofsfGFPperparticle=NR=CPP2/CPP1

$$\%\;\mathrm{free}\;\mathrm{cargo}=\left\{F1+\left(1-F1\right)*\left(1-\mathrm{NR}(t)/\mathrm{NR}(0)\right)\right\}*100$$

N is the effective number of diffusing particles in the confocal volume (N=n1+n2), $\tau_{D}$ is the diffusion time ($\tau_{1},\;\tau_{2}$ diffusion times of corresponding fractions), $F_{1}$ fraction of component with diffusion time $\tau_{1}$, whilst SP is the structural parameter defined as the ratio of height to width of the confocal volume (fixed to five). T is the triplet fraction with corresponding triplet time $\tau_{\mathrm{trip}}$. CPP is counts per particle in kHz. NR(0) is the number sfGFP per particle from the PBS data (one component fits above). NR(t) is the number of sfGFP per particle at timepoint *t* = 5.5 h. In a few cases, NR(t) was higher than NR(0) (a big particle or an aggregate measured), hence % of free sfGFP was set to 0 % (3 out of 75 total measurements).

The *x*‐*y* dimension of the confocal volume ($\omega_{\mathrm{xy}}^{2}$) was obtained through the calibration measurement with OG488 in PBS, using the known diffusion coefficient (D):

$$D=\frac{\omega_{\mathrm{xy}}^{2}}{4\tau_{D}}\;$$

Stokes–Einstein equation allowed subsequent determination of hydrodynamic radii ($R_{h}$) from the obtained diffusion coefficients (D). Control measurements of nonfluorescent EVs from HEK293T/17 cells did not yield any autocorrelation signal (average CR 0.2 kHz), as expected.

### 4.10 Immuno‐TEM

Copper grids with a continuous carbon ultrathin film (CF200‐Cu‐UL, Electron Microscopy Sciences) were O_2_‐plasma treated (30 s, 1 mbar; Plasma Prep 5, GaLa Instrumente, Germany) to enhance hydrophilicity. Grids were incubated on top of 10 μl droplets of EV suspension (G‐GFP EVs [3.17 × 10^11^ p/ml] or Native EVs [1.09 × 10^11^ p/ml]) for 20 min at RT. The samples were blocked with 5% BSA (AURION BSA‐c, 25557, Electron Microscopy Sciences) in PBS (15710, Electron Microscopy Sciences) for 20 min at RT and then washed with PBS. The samples were subsequently incubated with a rabbit polyclonal anti‐GFP antibody (1:25, ab6556, Abcam) in incubation buffer (0.1% BSA in PBS) for 1 h at 37°C. This was followed by incubation with goat antirabbit IgG (H&L) conjugated to 6 nm gold nanoparticles (25104, Electron Microscopy Sciences) for 20 min at 37°C. The immuno‐labelled samples were then washed in PBS and fixed in 0.1% PFA (15710, Electron Microscopy Sciences) in PBS for 20 min at RT. The samples were washed with Ultra‐Pure Distilled water (10977035, Thermo Fisher Scientific) before being negatively stained with 2% aqueous uranyl acetate solution for 15 s at RT. Following a final wash with water, the samples were air dried. Samples were examined via transmission electron microscopy (TEM) with a JEOL JEM‐2100Plus (JEOL, Japan) operated at an acceleration voltage of 200 kV. An objective aperture was placed to enhance contrast. Micrographs were acquired using an Orius SC1000 CCD camera (Gatan Inc., USA) at magnifications of 25,000× or 50,000×.

### 4.11 Magnetic bead‐based flow cytometry

Magnetic bead‐based flow cytometry was used to check the association of GFP with EVs, as previously described with modifications (Meyer et al., 2017). Ten micrograms SEC purified EVs were first incubated with 5 μl magnetic beads coated with either anti‐GFP, antihuman CD9, antihuman CD63 or antihuman CD81 for 1 h at room temperature with end‐to‐end rotation. Following incubation, the beads were washed three times with FACS staining buffer (BD Biosciences), and the GFP signal on the beads were evaluated on a BD LSRFortessa flow cytometer and data were analysed with Flowjo (v10.8), respectively. Anti‐GFP magnetic beads were generated by incubating protein A coupled Dynabeads (Thermo Fisher Scientific) with rabbit anti‐GFP (ab6556; Abcam) for 1 h at room temperature with end‐to‐end rotation. Anti‐CD9/63/81 magnetic beads were purchased from Thermo Fisher Scientific.

### 4.12 Immunofluorescence and confocal microscopy

The subcellular localization of GFP was determined by immunofluorescence, as previously described with modifications (Che et al., 2020). In brief, HEK293T/17 cells preseeded in an ibidi 8‐well plate (80827, ibidi) were first transfected with different GFP constructs for 24 h, and then cells were fixed with 4% PFA, and sequentially stained with WGA633 (membrane staining; 5 μg/ml, 200 μl; Thermo Fisher Scientific) and DAPI (nucleus staining; 1 μg/ml, 200 μl; Thermo Fisher Scientific) for 20 min each. Following washes with PBS, samples were imaged with confocal laser scanning microscopy (Leica SP5/MP).

### 4.13 Mice, ethical statement and vaccination

All animal experiments were performed in accordance with a project license (P63FE629C), and the authority of a personal license (ID7E0D627) granted by the UK Home Office under the Animals (Scientific Procedures) Act 1986. All protocols involving animals were reviewed and approved by the institutional Animal Welfare and Ethical Review Body (AWERB).

BALB/c mice (6–8 weeks old) were used in the current study. For plasmid DNA‐based immunizations, mice were injected twice with 10 μg DNA in 50 μl PBS i.m followed by electroporation (McKay et al., 2020). For protein or EV‐based immunizations, mice injected i.m with 50 μl of 2 μg purified soluble GFP (sfGFP), 2 μg sfGFP + 6 μg purified native EVs (EVs from untransfected HEK293T/17 cells), 8 μg G‐GFP‐EVs, or 8 μg H‐GFP‐EVs (EVs purified from G/H‐GFP transfected HEK293T/17 cells). The dose of 8 μg purified EVs was adopted from another similar immunogenicity mice study using purified bacteria‐derived EVs (Data not shown). All immunizations were performed twice in 4‐week intervals. Blood samples were taken at week 4 and 6, and mice were sacrificed at week 6. At each blood collection, 100 μl of peripheral blood was collected from the tail vein of each mouse.

### 4.14 ELISA

GFP‐specific IgG, IgA, IgG1, IgG2a and IgG2b were measured by semiquantitative ELISA, as previously described with modifications (McKay et al., 2020). In brief, ELISA plates (Nunc Maxisorp, Thermo Fisher Scientific) were first coated with purified GFP (test wells) or goat antimouse Kappa (1050‐01; Southern Biotech) and goat anti‐Lamda light chains (1060‐01; Southern Biotech) (standard curve) overnight at 4°C, and then washed with PBST and blocked with 1% BSA. Following another round of washes with PBST, plates were then incubated with serially diluted mouse sera samples or 5‐fold serially diluted Ig standards (standard curve) for 1 h at 37°C. For IgG, IgG1, IgG2a and IgG2b detection, plates were then incubated with HRP‐conjugated goat antimouse IgG (1030‐50; Southern Biotech), IgG1 (1073‐05; Southern Biotech), IgG2a (1083‐05; Southern Biotech) and IgG2b (1093‐05; Southern Biotech) respectively for another 1 h at 37°C. For IgA detection, plates were sequentially incubated with biotin‐conjugated goat antimouse IgA (1040‐08; Southern Biotech) and HRP‐conjugated Streptavidin (DY998; R&D systems) for 1 h and 30 min at 37°C, respectively. Following final washes, SureBlue TMB substrate (Insight Biotechnologies) was added to each well for colorimetric development, which was stopped by the addition of stop solution. Finally, plates were read at a testing wavelength of 450 nm and a reference wavelength of 800 nm on a Versamax Spectrophotometer (BioTek Industries). Antibody titers were calculated from the standard curve generated on each plate.

### 4.15 Luminex

At week 6, mice were sacrificed, and spleens were harvested and splenocytes were generated as detailed above. After washes with complete culture medium, splenocytes were stimulated in vitro with 10 μg/ml GFP, 5 μg/ml Con A (positive control) or medium (negative control) for 7 days at 37°C. Following incubation, cell culture supernatants were harvested and antigen‐stimulated Th‐related cytokines were measured by a Th1/Th2/Th9/Th17/Th22/Treg Cytokine 17‐Plex Mouse ProcartaPlex™ Panel (Invitrogen Thermo Fisher Scientific) on a BioPlex 200 (Bio‐Rad) platform, according to the manufacturers’ instructions.

### 4.16 Cell‐based flow cytometry

For EV in vitro uptake assay, splenocytes were isolated from naïve BALB/c mice and incubated with purified soluble sfGFP (2 and 10 μg), purified D‐GFP (10 μg), G‐GFP (10 μg) or H‐GFP EVs (10 μg) for 1 h at 37°C. After incubation, cells were washed, stained with LIVE/DEAD™ Fixable Aqua Dead Cell Stain Kit (Thermo Fisher Scientific) for 30 min at room temperature. Cells were then washed with FACS staining buffer and sequentially incubated with Mouse BD Fc Block (BD Biosciences) and fluorochrome‐conjugated antibodies for 5 min and 30 min at 4°C, respectively. The following antibodies were used: B cell (CD19‐BV605; 115540), T cell (CD3e‐BV711; 100349), DC (CD11c‐BV785; 117336), NK cell (CD49b‐APC/Cy7; 108920), macrophage (F4/80‐BV421; 123131), monocyte (CD11b‐PE; 101207), neutrophil (Ly6G‐BV650; 127641). Unless otherwise stated, all the fluorochrome‐conjugated antibodies were purchased from BioLegend. After final washes with FACS staining buffer, cells were fixed with BD Fixation buffer (BD Biosciences) for evaluation. The different immune cells were defined by the following markers: B cell (CD19+), T cell (CD3e+), DC (CD11c+CD3e‐CD49b‐), NK cell (CD49b+CD3e‐CD19‐), macrophage (F4/80+CD11c‐), monocyte (CD11b+CD3e‐CD19‐CD11c‐F4/80‐CD49b‐Ly6G‐) and neutrophil (Ly6G+).

For GFP in vivo expression analysis, BALB/c mice were first injected with 10 μg in 50 μl PBS of different GFP‐expressing plasmids or negative control plasmid pcDNA3.1 followed by electroporation. Twenty‐four hours post injection, mice were sacrificed, and injection site muscle was harvested, and single cell suspension was generated as described above. Following live/dead cell staining with LIVE/DEAD™ Fixable Aqua Dead Cell Stain Kit (Thermo Fisher Scientific) for 30 min at room temperature, cells were washed and resuspended in FACS buffer (BD Biosciences) for evaluation.

The evaluation was performed on a BD LSRFortessa flow cytometry and data analysis was performed with Flowjo (v10.8). Gating strategies were illustrated in Figure S11.

### 4.17 Statistical analysis

All data were expressed as mean ± standard deviation (SD) and all statistical analyses were performed with GraphPad Prism 8 (GraphPad). For comparisons between two groups, Mann–Whitney test was used. For comparisons among three or more groups, Kruskal–Wallis test plus Dunn's multiple comparisons test was used. A p value less than 0.05 was considered statistically significant.

## AUTHOR CONTRIBUTIONS

K.H., P.F.M. and R.J.S. designed the study. K.H., P.F.M., K.S., A.N., A.K.B., J.C., G.O. and M.C. performed experiments, aided by M.M.S. K.H. and A.N. analysed the data. K.H. wrote the first draft of the manuscript. P.F.M., A.N., A.K.B. and R.J.S. edited the manuscript with constructive comments from other authors.

## CONFLICT OF INTERESTS

The authors declare that no competing interests exist.

## Supporting information

Supporting Information
