# Supplementary material for: Presentation of antigen on extracellular vesicles using transmembrane domains from viral glycoproteins for enhanced immunogenicity
Source: J Extracell Vesicles. 2022 Mar 1;11(3):e12199. doi: 10.1002/jev2.12199 (PMC8888812; doi:10.1002/jev2.12199)
Supplement: Supplementary file 1 — Supporting Information [file JEV2-11-e12199-s001.docx]

Supplementary Materials

**Supplementary Table 1. List of GFP constructs designed in this study.**

| Name | Design |
| --- | --- |
| A-GFP | sfGFP with the secretion signal peptide from rat follicle stimulating hormone beta-subunit. |
| B-GFP | sfGFP without secretion signal peptide. |
| D-GFP | A-GFP followed by a (G_4_S)_2_ linker and VSV-G TM and CT. |
| E-GFP | A-GFP followed by a (G_4_S)_2_ linker and RABV-G TM and CT. |
| F-GFP | A-GFP followed by a (G_4_S)_2_ linker and PIV5-G TM and CT. |
| G-GFP | A-GFP followed by a (G_4_S)_2_ linker and MARV-G TM and CT. |
| H-GFP | A-GFP followed by a (G_4_S)_2_ linker and ZEBOV-G TM and CT. |
| I-GFP | A-GFP followed by a (G_4_S)_2_ linker and Lassa-G TM and CT. |
| J-GFP | A-GFP followed by a (G_4_S)_2_ linker and H3-Udorn TM and CT. |
| K-GFP | A-GFP followed by a (G_4_S)_2_ linker and HIV 750 Env TM and CT. |

**Supplementary Table 2. Forms of GFP produced by different GFP constructs.**

|  | Construct | A-GFP  (sGFP) | B-GFP  (in-cell) | D-GFP  (VSV) | G-GFP  (MARV) | H-GFP  (ZEBOV) |
| --- | --- | --- | --- | --- | --- | --- |
| GFP form | soluble | √ |  |  |  |  |
|  | intracellular | √ | √ | √ | √ | √ |
|  | cell surface | √ |  | √ | √ | √ |
|  | EV surface |  |  | +^*^ | ++^*^ | ++^*^ |

Note:

*: G-GFP and H-GFP produce more EV-bound GFP than D-GFP.


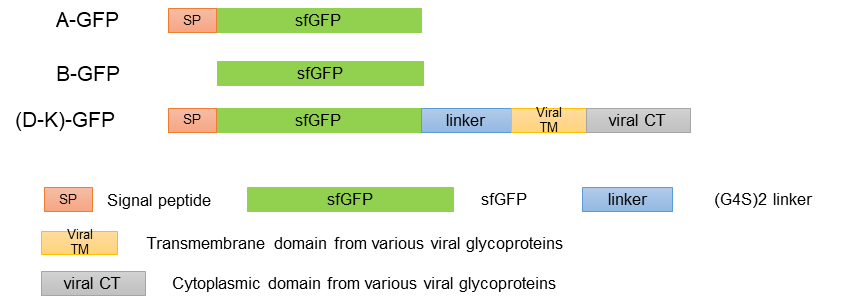


**Figure S1.** Design of the different GFP constructs. A-GFP was designed by attaching a secretion signal peptide from rat follicle stimulating hormone beta-subunit to the N terminus of sfGFP. B-GFP was the original coding sequence of sfGFP. (D-H)-GFP constructs were designed by adding a (G4S)2 linker followed by TMs and CTs from VSV-G (D-GFP), RABV-G (E-GFP), PIV5-F (F-GFP), MARV-G (G-GFP), ZEBOV-G (H-GFP), Lassa-G (I-GFP), H3-Udorn (J-GFP) and HIV 750 Env (K-GFP) to the C terminus of A-GFP.


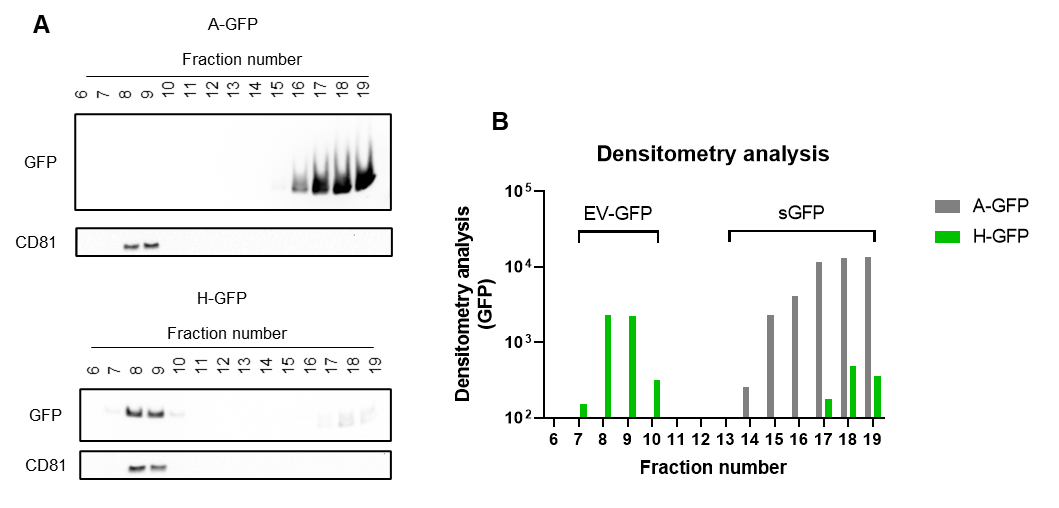


**Figure S2.** EV purification by SEC. EVs from cell culture supernatant were first purified by qEVoriginal/70nm SEC columns and 20 of 0.5 mL fractions were collected. The fractions were then concentrated with 10kD Amicon ultrafiltration tubes and the presence of GFP and CD81 in the fractions were determined by Western blot (A). The relative protein concentration in the fractions were then semiquantified by densitometry analysis of the Western blot bands using Image J (B).


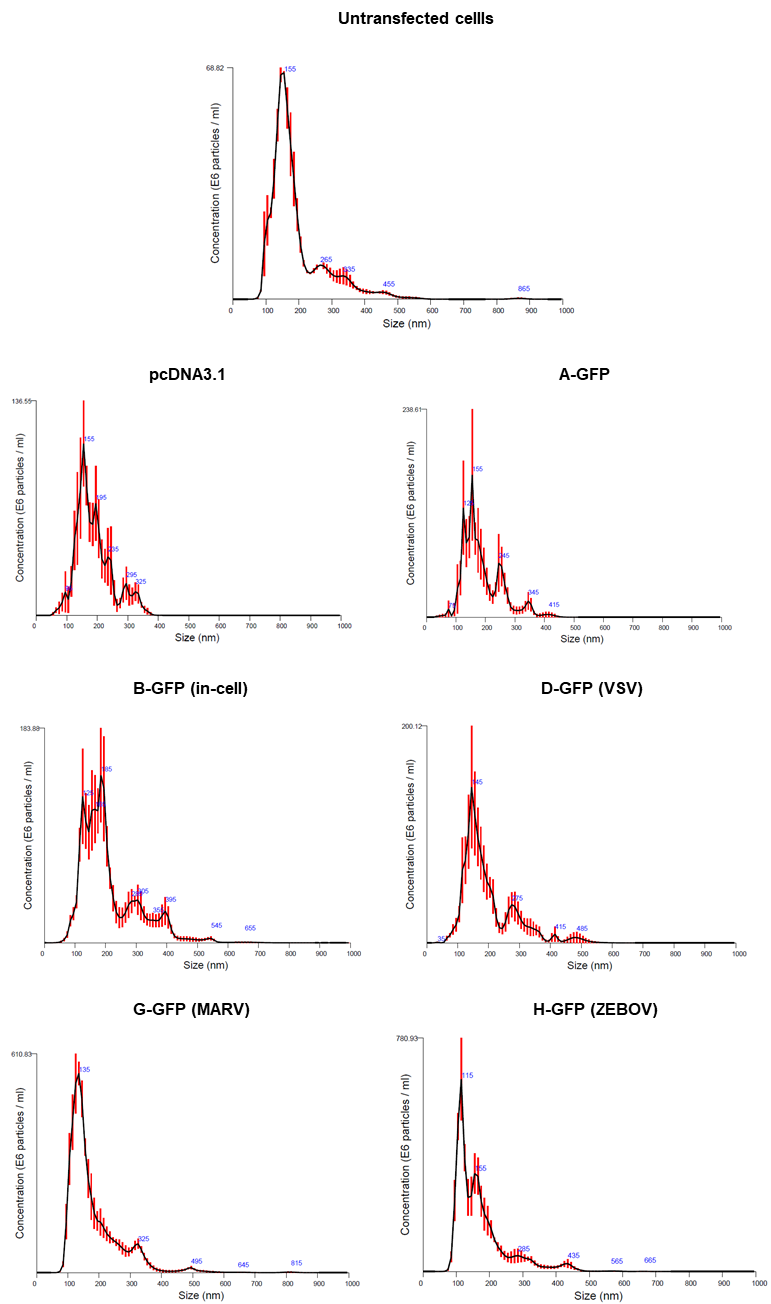


**Figure S3.** NTA analysis of purified EVs. EVs were first purified by SEC and then the size distribution was determined by NTA with a Nanosight LM10 machine. For each sample, 3 videos of 60s each were taken, and the averaged size distribution was calculated and graphed.


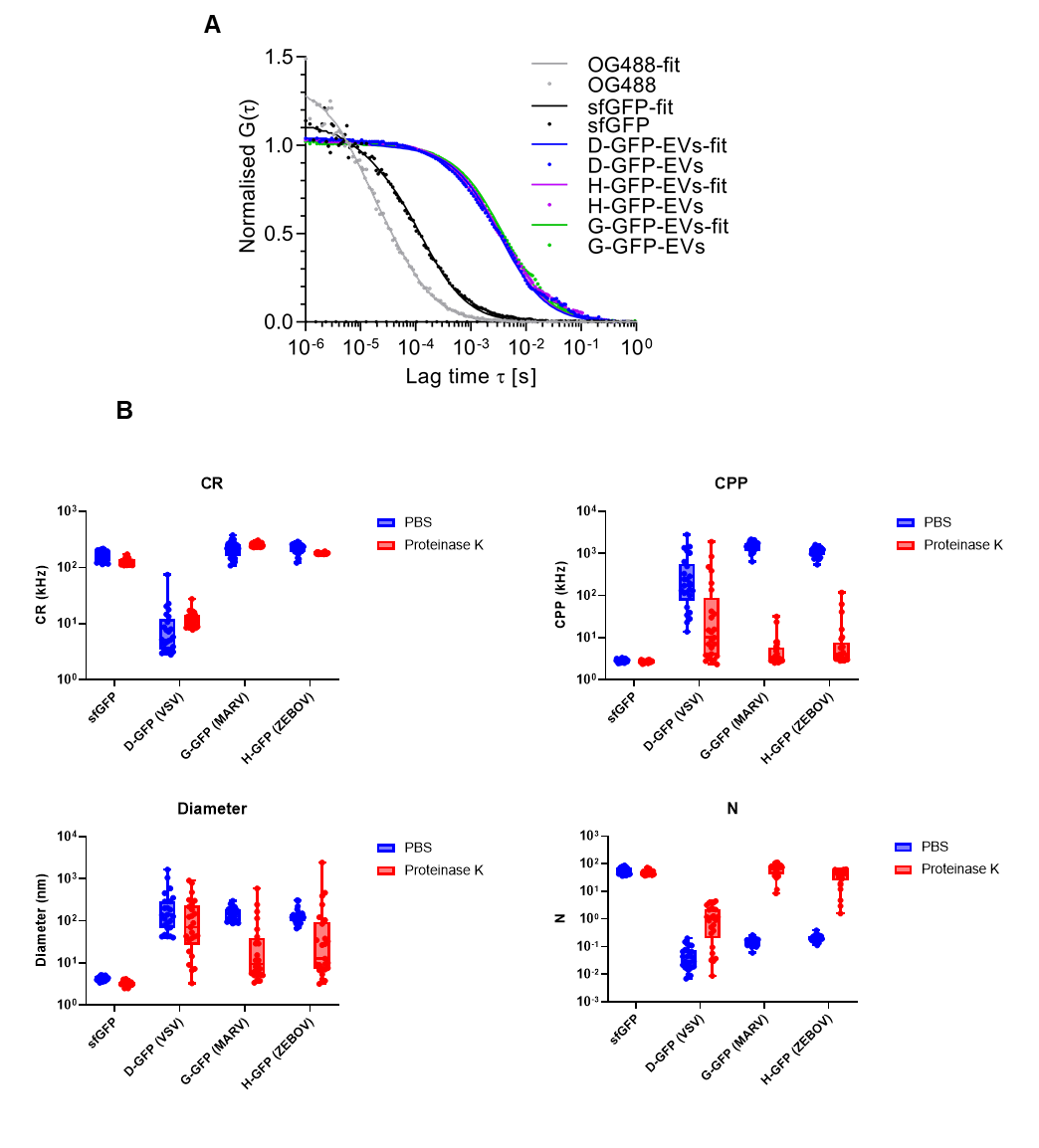


**Figure S4.** FCS analysis of purified EVs. Purified sfGFP (300 µg/mL) or EV samples (1 × 10^11^ particles/mL) were first incubated with PBS or proteinase K in PBS, and then FCS measurements were recorded (free GFP was diluted 100x before measurements and EVs were measured undiluted). Oregon Green 488 (OG488) was used as a standard for beam calibration. Intensity traces of 25×5 or 10 s were recorded for each sample and the intensity fluctuations were autocorrelated and analyzed. (A) Autocorrelation curves (dots) and corresponding fits (lines) were shown. (B) CR, CPP, diameter and N of each sample with or without proteinase K treatment were shown.


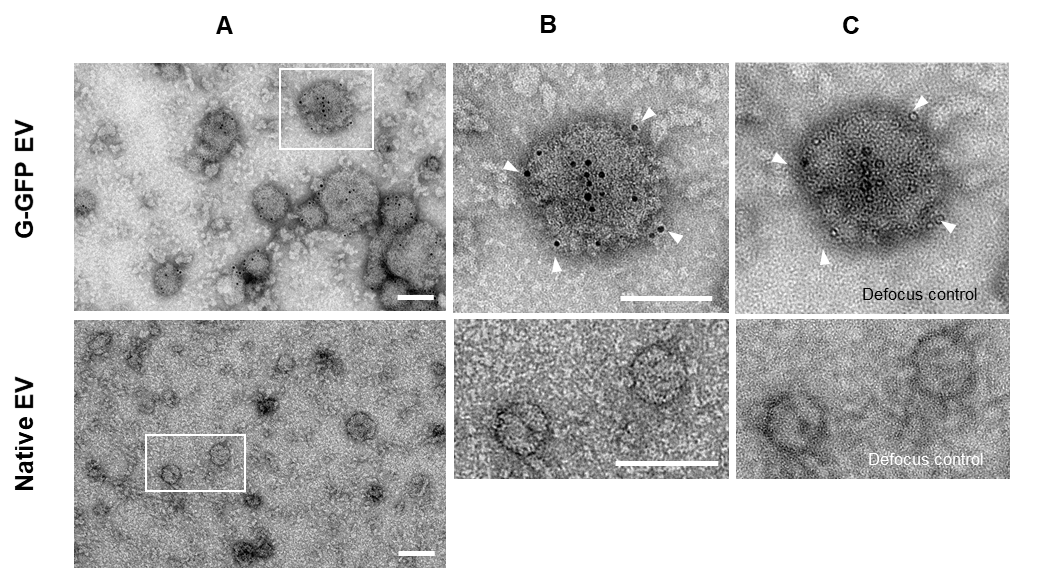


Figure S5. TEM micrographs of EVs. (A) TEM micrographs showed the size dispersion and consistent presence (G-GFP EVs) or absence (native EVs) of immunogold labelling for GFP. (B) Inset to (A) showing individual EVs. The area in (B) corresponds to the white-framed area marked in (A). (C) Micrograph taken at an overfocus, showing the typical change in contrast of gold nanoparticles (G-GFP EVs). Scale bars represent 100 nm. Images in (C) are the same scale as their corresponding images in (B).


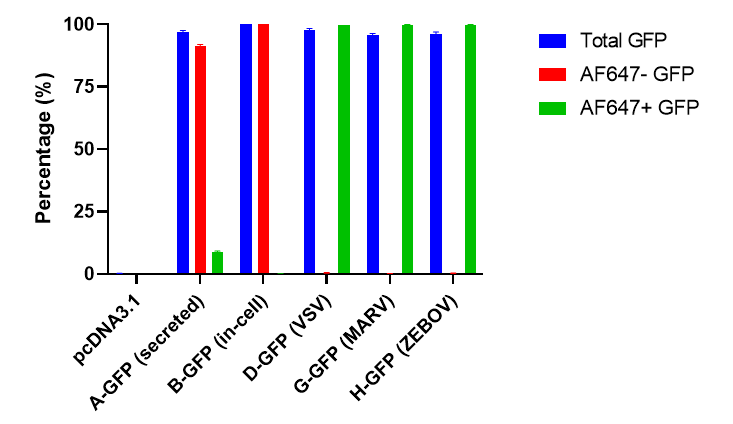


**Figure S6.** Subcellular distribution of GFP in transfected cells. HEK293T/17 cells were transfected with different GFP plasmids for 24 h and then cell surface GFP was stained with anti-GFP-AF647 and GFP and AF647 signal were recorded by flow cytometry. Data shown are mean ± SD of three independent experiments.


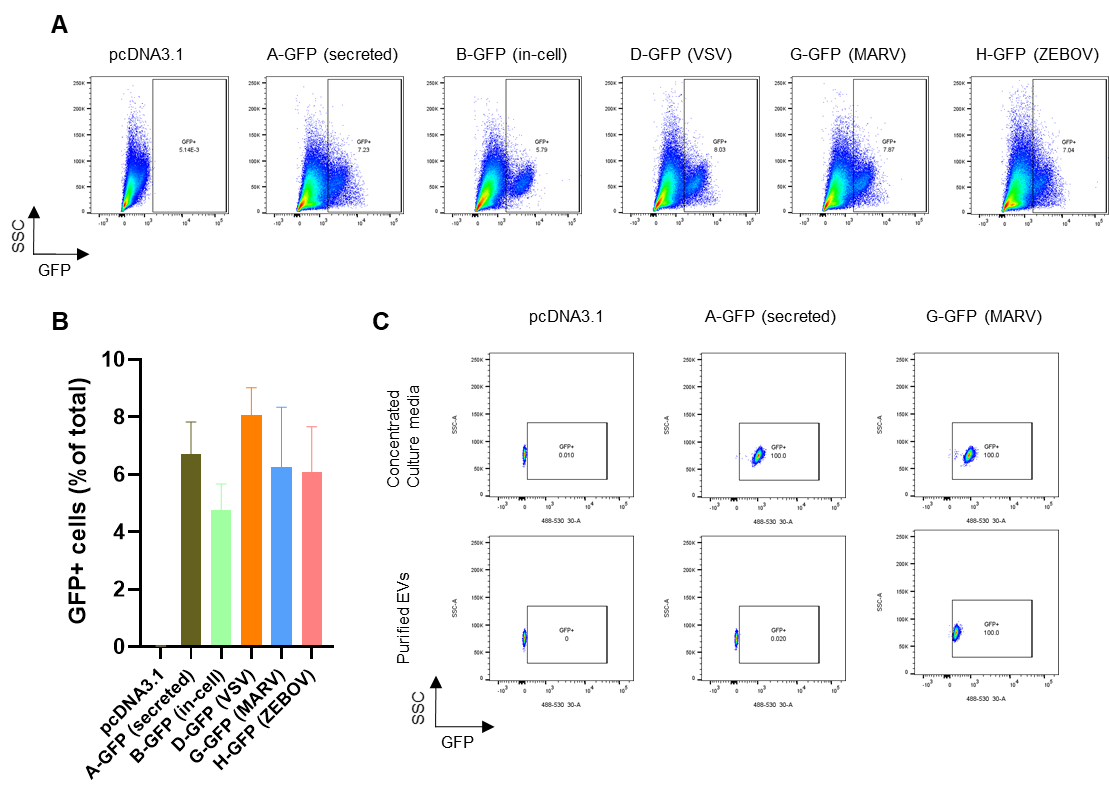


**Figure S7.** Viral TM-bearing GFP plasmids produce GFP EVs in vivo. Mice were injected with different GFP plasmids for 24h and then injection site muscle samples were isolated and single cell suspension was generated and (A-B) GFP+ cells were quantified by flow cytometry. (A) One representative result is shown. (B) Data shown are mean ± SD of 4 injection sites. (C) Isolated muscle cells were cultured in skeletal muscle growth medium for 3 days and then EVs were purified from the culture medium by SEC and GFP signal in the purified EVs or concentrated medium was measured by anti-GFP beads-based flow cytometry. One representative data out of 3 is shown.

**Figure S8.** EV-GFP-producing plasmids enhance antigen-specific cell-mediated responses. Mice were first immunized with different GFP plasmids twice and 2 weeks after the second injection, mice were sacrificed and splenocytes were prepared and restimulated with purified GFP for 7 days. Following stimulation, cytokines released by the splenocytes were quantified by a Th1/Th2/Th9/Th17/Th22/Treg Cytokine 17-Plex Mouse ProcartaPlex™ Panel (n=5).

**Figure S9.** EV-GFP enhances antigen-specific cell-mediated responses. Mice were immunizaed with either purified sfGFP, sfGFP+EV, G-GFP EV or H-GFP EV twice in 4-week intervals. Two weeks after the second injection, mice were sacrificed and splenocytes were prepared and restimulated with purified GFP for 7 days. Following stimulation, cytokines released by the splenocytes were quantified by a Th1/Th2/Th9/Th17/Th22/Treg Cytokine 17-Plex Mouse ProcartaPlex™ Panel (n=5).


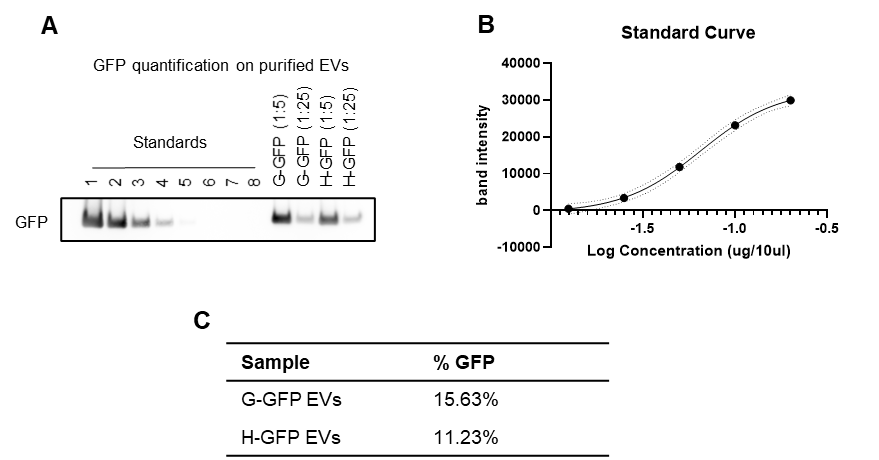


**Figure S10.** Quantification of GFP content on EVs. (A) GFP signal in diluted G-GFP and H-GFP EVs as well as 2-fold serially diluted purified GFP standards was detected by western blot. (B) The band intensity was quantified by Image J and a standard curve of GFP concentration against band intensity was generated by 4-parameter logistic regression in GraphPad Prism 9 and (C) GFP content in G-GFP and H-GFP EVs were calculated off the standard curve. Goodness of fit: r^2^ = 1.000.


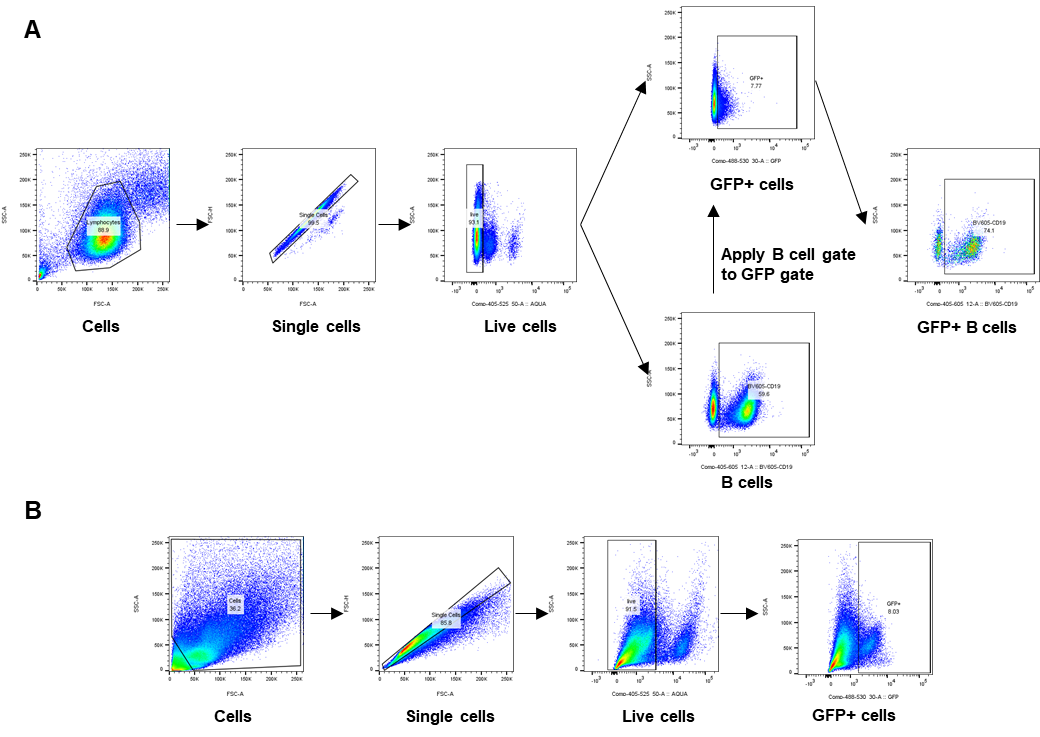


**Figure S11.** Gating strategy for GFP+ cell subset identification. (A) Gating strategy for identification of GFP+ cell subsets in splenocytes. (B) Gating strategy for GFP+ cells in mice muscle.


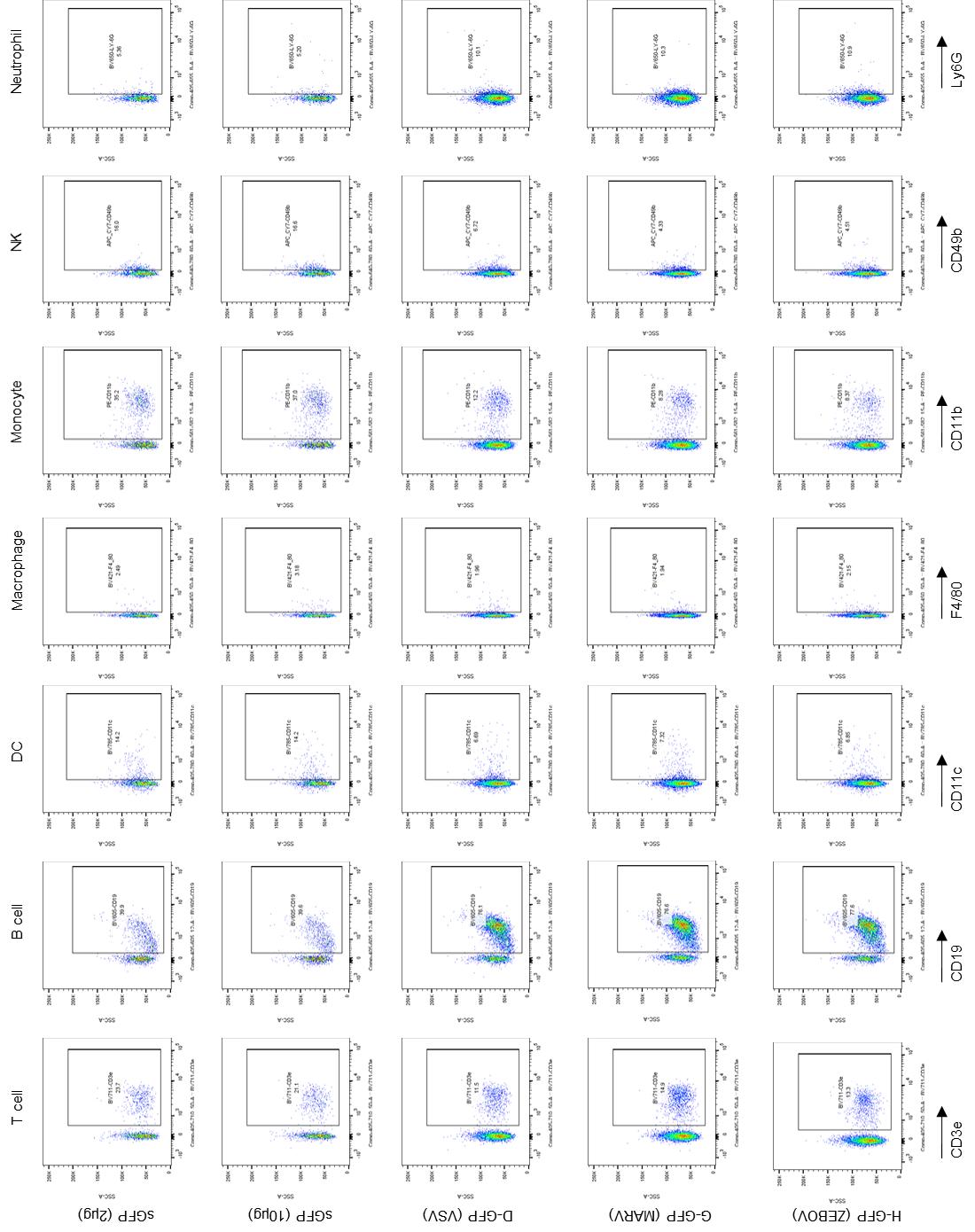


**Figure S12.** Dot plots of GFP+ cell subsets. Cells were stained with the following cell subtype markers B cell (CD19-BV605), T cell (CD3e-BV711), DC (CD11c-BV785), NK cell (CD49b-APC/Cy7), macrophage (F4/80-BV421), monocyte (CD11b-PE) and neutrophil (Ly6G-BV650), and GFP+ cell subtypes were analyzed. The dot plots showed the cell subset distribution of total GFP+ cells. One representative result is shown.

**Figure S13.** GFP shedding from EV/cell surface. HEK293T/17 cells were first transfected with B-GFP (in-cell) or E-GFP (RABV) plasmids for different times, and then GFP expression in the cell culture supernatant and cell lysate was determined by Western blot. A representative result from three independent experiments is shown.
